# Amplatzer Patent Foramen Ovale Occluder Device-Related Complications

**DOI:** 10.7759/cureus.23756

**Published:** 2022-04-02

**Authors:** Beshoy Iskander, Fatima Anwer, Federico Oliveri, Kakargias Fotios, Priyanka Panday, Ana P Arcia Franchini, Pousette Hamid

**Affiliations:** 1 Internal Medicine, California Institute of Behavioral Neurosciences & Psychology, Fairfield, USA; 2 Neurology, California Institute of Behavioral Neurosciences & Psychology, Fairfield, USA; 3 Cardiology, California Institute of Behavioral Neurosciences & Psychology, Fairfield, USA; 4 Medicine, California Institute of Behavioral Neurosciences & Psychology, Fairfield, USA; 5 Research, California Institute of Behavioral Neurosciences & Psychology, Fairfield, USA

**Keywords:** pfo, pfo closure, amplatzer plug device, amplatzer occluder, atrial fibrillation, amplatzer, percutaneous pfo closure, pfo closure complications, pfo occluder device, residual shunt

## Abstract

Patent foramen ovale (PFO) is a standard variant that is present in 25% of the whole adult population. In a certain population, PFO can lead to cerebrovascular accidents. Mechanism of cerebrovascular accidents can be by paradoxical embolization from the right circulation or in situ thrombosis. Diagnosis of a PFO-responsible cerebrovascular accident is based on a thorough work-up to exclude other possible etiologies and detect PFO on trans-thoracic or trans-esophageal echocardiography with bubble study and/or Doppler. Over the last few years, multiple studies have supported that percutaneous PFO closure is superior to medical therapy in the secondary prevention of cerebrovascular accidents. However, numerous adverse events have been linked to PFO closure devices in general compared to medical therapy as new-onset atrial fibrillation, residual shunt, device-related thrombus, bleeding, deep vein thrombosis, pulmonary embolism, and inter-atrial septal erosions. Amplatzer device is one of the PFO occluder devices approved by the FDA. Device-related adverse events have been addressed by comparing the Amplatzer device with other PFO occluder devices. Based on the new data, we expect to see more complications related to PFO closure in the coming few years. We reviewed different studies that looked at the PFO closure-related complications and the trials comparing adverse events in the Amplatzer PFO occluder device compared to other devices. Amplatzer PFO occluder device is either superior or non-statistically different from other PFO occluder devices related to new-onset atrial fibrillation and residual shunt. More studies are needed to address the other less common adverse events. Since many of the device-related complications appear many years after device placement, a long-term follow-up is recommended.

## Introduction and background

Patent foramen ovale (PFO) is a normal variant of the atrial septum and is considered a remnant of normal fetal anatomy. At six months, more than half of the infants will have a PFO. In adults, PFO is often asymptomatic [1,2]. PFO is usually present in about 25% of the adult population [3]. PFO is considered responsible for about 30-40% of young patients having a cryptogenic stroke [3-6]. Generally, the risk of cryptogenic strokes increases with a larger defect and with the presence of an interatrial septal aneurysm. PFO can be classified based on size by measuring the maximum opening between septum primum and secundum to large (>4 mm), medium (2-3.9 mm), and small (<2 mm) [7,8]. PFO in some adults can lead to paradoxical embolism, decompression sickness, migraine, and platypnea orthodeoxia [1,2]. The risk of paradoxical embolization is higher with larger defects and the presence of an interatrial septal aneurysm. These factors predispose to embolization by increasing the risk of in situ thrombosis and septal aneurysms associated with larger defects [3-6].

Treatment of PFO has always been challenging on whether to follow medical treatment (including single antiplatelet, dual antiplatelet therapy [DAPT], or anticoagulation) or PFO closure. Initial randomized clinical trials (CLOSURE, RESPECT, and PC trial) did not show a statistically significant superiority in treating PFO patients with closure devices vs. medical therapy [9-12]. Recent clinical trials (CLOSE, CORE REDUCE, DEFENSE PFO) and the extended follow-up study of the RESPECT clinical trial have shown a statistically significant superiority of the PFO closure compared to medical therapy [10-14]. The Amplatzer PFO occluder device has a significantly lower recurrent stroke risk on the sensitivity analysis of three trials that used the device when compared to medical therapy [15].

**Figure 1:**
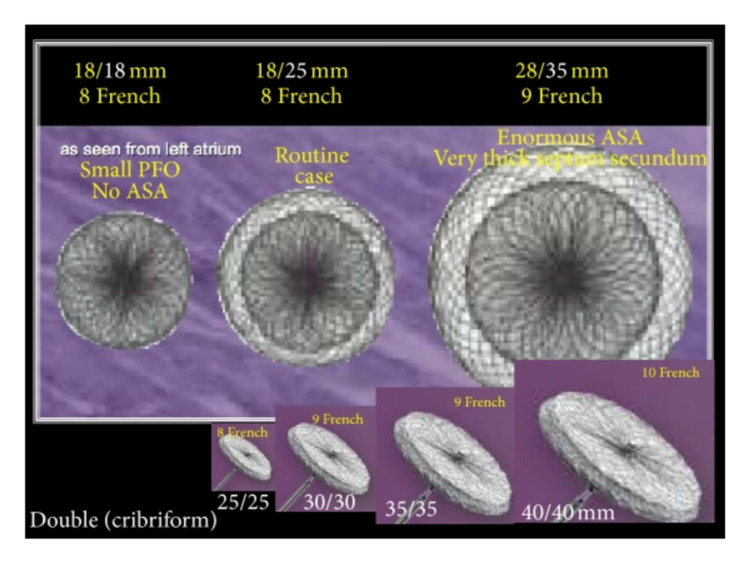
Amplatzer patent foramen ovale occluder in different sizes Source: Ref. [16]. Permission to use this figure was obtained from Hindawi (Scientifica).

The Amplatzer PFO occluder device was first introduced in 1997 by Kurt Amplatzer [16]. The Amplatzer occluder (Figure 1) is made of an alloy of nickel and titanium, shaped into two flat discs and a middle to fit the defect size. It is associated with polyester fabric inserts to help in the defect closure and to provide a space for tissue growth over the occluder after its placement [17]. The device was approved by the American Food and Drug administration in 2016 to treat PFO to prevent recurrent strokes in patients who had a cryptogenic stroke. The complications reported following an Amplatzer device placement for PFO closure are limited.

In this article, we will highlight and review the available data on the complications associated with PFO closure with an Amplatzer device.

## Review

We will analyze several studies, including systematic review, meta-analysis, randomized controlled trials, observational studies, and case reports, found in the PubMed database and Google Scholar database. Keywords used in this review include PFO, PFO occlusion, PFO occluder device, septal erosion, Gore, Cardioseal, STARflex, and Amplatzer. Inclusion criteria include the articles published in the last 20 years, PFO, and an indication for PFO closure. Exclusion criteria included animal and duplicated studies. Fifty-four studies resulted, and the findings from 45 relevant studies were included in our review.

New-onset atrial fibrillation

The incidence of atrial fibrillation after PFO device closure is reported to be 0.5% after PFO occluder device implantation [10]. Supraventricular tachycardia (SVT) after PFO closure is due to the change in the right atrium and left atrium size after the closure and geometric changes in the left atrium [18]. Multiple studies have looked at the incidence of atrial fibrillation and the comparison of Amplatzer occluder devices to other PFO occluder devices [19-30]. In the RESPECT trial, when comparing medical therapy to percutaneous PFO closure with the Amplatzer PFO occluder, the rate of atrial fibrillation was not statistically different in the medical treatment group and the intervention group (OR, 1.47; 95% CI, 0.64-3.37; p-value, 0.36) [11]. In the trial, the cryptogenic stroke was defined as a stroke from an unknown cause according to the supplementary index. According to the study protocol, a cardiac monitor was recommended before the diagnosis only if atrial fibrillation is suspected and is not a standard procedure, which may overestimate the atrial fibrillation incidence following device implantation [11]. In the Close Trial, new-onset atrial fibrillation was detected early on, in one month, after device implantation and not on the follow-up period, which would suggest that the atrial fibrillation might have been induced by manipulation and device-related inflammation and might not be a long-term complication [13].

Multiple meta-analysis studies supported that compared to the medical therapy, the intervention group was having a statistically significantly increased risk of atrial fibrillation and SVT after device implantation [19-30]. A meta-analysis of three randomized clinical trials, Closure I, PC Trial, and RESPECT, including non-specified device types, Amplatzer, Gore Helex, and Gore septal occluder device, respectively, when compared to antiplatelet medical therapy or anticoagulation showed that there is a statistically significant increase in the atrial fibrillation in the intervention group (OR, 3.77; 95% CI, 1.44-9.87; p-value, 0.007) [19].

Capodanno et al. reported that the new-onset atrial fibrillation was observed more frequently in the STARflex/Cardioseal occluder device when compared to the Amplatzer PFO occluder device (95% CI, 50.18-0.48) in the meta-analysis of 20 studies from 2001 to 2011 [19]. The meta-analysis study included PFO and ASD occluder devices; one of these studies included patients less than five years of age, which might have confounded the result [19]. On the other hand, in Ntaios et al.'s subgroup analysis study of different PFO occluder devices used in PC, RESPECT, and Closure-I trials, the Starflex PFO occluder device was thought to have driven the negative results of the Closure-I trial for the new-onset atrial fibrillation [21]. New-onset atrial fibrillation was similar in the medical therapy and the Amplatzer PFO occluder on the subgroup study analysis (OR, 1.81; 95% CI, 0.60-5.42) as opposed to the Starflex occluder (OR, 8.30; 95% CI, 2.47-27.84) [22]. Similarly, Capodanno et al., on a meta-analysis of randomized controlled trials and observational studies, reported a similar outcome with higher estimates of new-onset atrial fibrillation for Starflex occluder compared to Amplatzer (OR, 9.20; 95% CI, 2.7-30.9; p-value, <0.001 vs. OR, 2.25; 95% CI, 1.03-4.94; p-value, 0.04) [22]. Also, Stortecky et al. reported a similar outcome of meta-analysis of Closure-I, RESPECT, and PC trials and Hornung et al. [23]. Palaidimos et al. on a systematic review and meta-analysis of Closure-I, PC, RESPECT, Gore Reduce, and Close trials reported that the Amplatzer device did not show a statistically significantly increased risk of atrial fibrillation/flutter (RR, 2.11; 95% CI, 0.80-5.56) [27]. On the other hand, Fortuni et al. reported a marginally increased risk of atrial fibrillation of Amplatzer PFO occluder device subgroups, including eight studies with a mean follow-up period of 2.8 years [28].

In summary, following PFO closure, the incidence of new-onset atrial fibrillation is more likely to be device-dependent according to many studies with the Amplatzer PFO occluder showing superior results in many of them. The interplay between the diagnosis of cryptogenic stroke and the incidence of atrial fibrillation following PFO closure yet might be contaminating the incidence of events; however, they yet showed a statistically significant increase in multiple meta-analysis articles.

Residual shunt

Residual shunt after PFO closure increases the risk of stroke via paradoxical embolization. Residual shunt in the first few months after successful percutaneous PFO closure is expected in different devices used [19,31-33]. In Deng et al.'s prospective cohort study on 1078 patients who underwent PFO closure secondary to a cryptogenic stroke to study residual shunt and the attributable risk of stroke, the patients were followed for a mean of 3.7 years. A residual shunt was observed in 22.5% of 243 patients, of which 13.9% are small shunts and 8.6% moderate to large; the patients' characteristics, medications, and comorbidities were not statistically different in the shunt and the no shunt groups [31]. The primary outcome of the study was a composite score of transient ischemic attack (TIA) and stroke that occurred 2.32 times in 100 patients in the shunt group and 0.75 times in 100 patients in the no shunt group with a risk of stroke or recurrent TIA (HR, 3.05; 95% CI, 1.65-5.62; p-value, <0.001) [31]. There was also an increased risk of TIA and stroke with moderate and large shunts when compared to small shunts as increased shunt size was associated with increased stroke or TIA recurrence (HR, 2.11; CI, 1.48-3.01; p-value, <0.001) [32]. Four PFO occluder devices were used: Amplatzer (n=700), StarFlex (n=225), HELEX (n=100), and Sideris button (n=53). The risk of the residual shunt was significant in HELEX and Sideris button groups only, but no statistically significant difference in the risk of the primary composite endpoint of recurrent TIA and stroke was found in all four devices [31]. Early residual shunt (within one month after closure) was assessed in six studies, including 1011 patients; when comparing the two devices (Amplatxer and CARDIOSeal/STARflex), there was no significant difference in the rate of an early residual shunt [19]. Middle-term residual, defined as a residual shunt within one to six months after PFO occluder device placement, was studied in 673 patients in six studies; when comparing the two devices, there was a significant difference [19]. Long-term residual shunt, defined at a time interval of six months to 24 months, evaluating 544 patients in four studies showed a statistically significantly lower risk rate in the Amplatzer PFO occluder group when compared to the CardioSeal/STARflex group (OR=0.37, 95% CI=0.14-0.97, p=0.04) [19]. 

Factors that increase the risk of the residual shunt include atrial septal aneurysm and the longitudinal dimension of the PFO at 12 months [32]. Hammerstring et al. reported a study comparing Amplatzer, Cardiodeal, Helex, and premere occluders in a total of 124 patients [33]. Closure rates were compared in the four devices with the highest closure rate reported with the Amplatzer device at 3-, 6-, 12-, and 24-month intervals [33]. The use of Helex device (HR, 12.58; 95% CI, 2.57- 57.43; p-value, 0.002), PFO-canal-length per mm (HR, 1.17; 95% CI, 1.05-1.31; p-value, 0.004), and the extent of atrial septal aneurysm (HR, 1.13; 95% CI, 0.95-1.29; p-value, 0.05) were determined as independent predictors for interatrial shunt after successful percutaneous PFO closure [33]. 

In summary, the type of the PFO occluder device used, the extent of the PFO, and the presence of interatrial septal aneurysm increase the risk of residual shunting after PFO closure. The more the shunt burden increases, the more the likelihood of TIA and stroke. 

Thrombus formation

Device-related thrombus formation following percutaneous PFO closure was described in various clinical trials with an incidence of <1% across all device types [12,13]. When it occurs, it leads to damage of the device, potential need for surgical removal, and risk for systemic and pulmonary embolization [19]. Device-related thrombus tends to be early after device placement and rarely occurs after 12 months [34-37]. Most of the PFO occluder devices are composed of metal (usually Nitinol) and polyester mesh, including the Amplatzer device. Coagulation within the wire mesh leads to and promotes endothelialization and complete closure of the defect. According to the randomized controlled trials including CLOSE, CLOSURE, DEFENSE PFO, and RESPECT trials, post-procedural management of DAPT for the first six months after the procedure and aspirin thereafter have been used in practice to lower the thrombotic risks after the device implantation, which usually occurs early on after the implantation rather than later in the course [10-14].

Li et al., in a meta-analysis study comparing the Amplatzer PFO occluder to the Cardioseal involving 1221 patients from seven studies, reported a significantly lower rate of thrombus formation in the Amplatzer group (OR, 50.07; 95% CI, 0.02-0.21; p-value, <0.00001) [19]. In a study analyzing data from 2303 patients in three randomized clinical trials and 2231 patients from 11 observational studies when pooling data where Amplatzer device was used in comparison with STARflex device, STARflex was associated with an increased risk of cardiac thrombus [22].

In summary, the device-related thrombus is a dangerous complication following PFO occluder device placement that necessitates starting DAPT. It is most likely to occur in the first few months after device implantation. In the reviewed articles Amplatzer PFO occluder device showed superiority in terms of a lower prothrombotic effect compared with other devices.

Bleeding

Bleeding in PC, ClOSURE, RESPECT, Reduce, Close, and Defense trials had different definitions [10-14]. It included a broad spectrum of definitions: gastrointestinal bleeding, severe or fatal bleeding, or even hemorrhagic stroke. Yet, in all these trials, bleeding events did not show any statistically significant difference [10-14]. In a meta-analysis of the six trials, bleeding was not shown to be statistically significantly different in the PFO closure group when compared to medical therapy (RR, 0.91; 0.60-1.38, p=0.66) [29]. According to our review, no studies were found addressing the bleeding risk in Amplatzer PFO occluders compared to other PFO occluders. In summary, PFO closure does not seem to increase the bleeding risk, according to the studies reviewed. 

Deep vein thrombosis/pulmonary embolism

Deep vein thrombosis (DVT) and pulmonary embolism (PE) can occur after PFO closure either as a coincidence or as a complication of device-related right atrial thrombus. In the randomized controlled trials, when comparing PFO closure to medical therapy, no strict criteria were mentioned about DVT or PE before enrolling in the studies. Also, DVT and PE after PFO closure were reported as adverse events following PFO closure in the GORE REDUCE, CLOSE, and DEFENSE PFO trials with no protocol-based investigation after the procedure [10-14]. Among the landmark trials on PFO closure, only the RESPECT trial showed a statistically significant increase of DVT/PE in the PFO closure group compared to the conservative medical group. No head-to-head studies were found comparing different PFO occluder types according to our review.

Atrial septal erosion

Atrial septal erosion is an infrequent complication following percutaneous PFO closure. Reported event rate is <0.1% [38-43]. It tends to occur in the first 72 hours following implantation, with few reported cases of late presentation [38-43]. Most of the reported cases of atrial septal erosion occurred in the anterosuperior border, increasing the risk of aortic perforation, pericardial diffusion, and immediate need for surgical intervention [38-43]. Due to the rarity of the event, no head-to-head studies comparing the Amplatzer PFO occluder device to other devices were found in our review. McElhinney et al. reported multiple risk factors that increase the risk of device erosions in atrial septal defect Amplatzer occluder devices [43]. No studies were reported to address the possibility of higher risk in PFO occluder devices, possibly due to the rarity of the events.

Anticoagulation

The use of anticoagulation after PFO closure is an area of debate. The CLOSE trial could not determine if there is any benefit compared to antiplatelet agents [13]. The use of DAPT for several months after percutaneous PFO closure followed by appropriate long-term aspirin was studied in many trials [10-14]. Anticoagulation has a rule in treating device-related thrombus and modalities, including surgical removal and lytic therapy [34]. Anticoagulation with certain PFO occluders has not been studied in head-to-head trials.

Procedural complications 

Device embolization was not statistically different when comparing the Amplatzer device to the CardioSEAL/STARFlex device and was overall rarely reported [19,33]. Air embolization and pericardial effusion were reported with the CardioSEAL and Amplatzer devices [33]. Overall procedural-related complications were a few separate cases with no study supporting that particular device implantation has an increased risk of occurrence of procedural complications.

Limitations

Our study was limited by the rarity of the adverse events of percutaneous PFO closure. Also, only a few studies compared the Amplatzer PFO occluder to other devices from a safety profile standpoint.

## Conclusions

Most of the complications following percutaneous PFO closure are rare. Based on the new data highlighting the evidence on PFO closure in secondary prevention of stroke compared to medical therapy with antiplatelet, we expect to see more device implantation in the future, which further necessitates the importance of identifying various device-related complications. Amplatzer PFO occluder device showed superior or non-statistically significant differences in terms of the risk of new-onset atrial fibrillation, residual shunt, and device-related thrombus when compared to different PFO occluder devices. Due to the rarity of device-related bleeding, DVT, PE, and atrial septal erosion, no direct studies were found comparing Amplatzer PFO occluder devices to other devices. We recommend an extended follow-up trans-thoracic echocardiography to detect and manage long-term complications following PFO device closure. We also suggest more clinical studies comparing adverse events and the efficacy of different PFO occluder devices. Also, more studies are needed to address the risk factors for the emergence of such adverse events. More questions than answers seem to be present based on our review when it comes to device selection.
